# Variability and longitudinal dynamics of donor-derived cell-free DNA in kidney and liver recipients: a comparison of absolute and relative quantities in plasma and urine

**DOI:** 10.3389/frtra.2026.1790754

**Published:** 2026-06-02

**Authors:** Fanny Sandberg, Nicholas Kueng, Vanessa Banz, Annalisa Berzigotti, Daniel Sidler, Carlo R. Largiadèr, Ursula Amstutz

**Affiliations:** 1 Graduate School for Cellular and Biomedical Sciences, University of Bern, Bern, Switzerland; 2 Department of Clinical Chemistry, Inselspital, Bern University Hospital, University of Bern, Bern, Switzerland; 3 Department of Visceral Surgery and Medicine, Inselspital, Bern University Hospital, University of Bern, Bern, Switzerland; 4 Department of Nephrology and Hypertension, Inselspital, Bern University Hospital, University of Bern, Bern, Switzerland

**Keywords:** droplet digital PCR (ddPCR), donor-derived cell-free DNA, kidney transplantation, liver transplantation, plasma, urine

## Abstract

**Background:**

Donor-derived cell-free DNA (ddcfDNA) is increasingly being integrated into clinical practice for detecting allograft rejection in solid organ recipients. Natural fluctuations in ddcfDNA or other conditions can affect interpretation. Therefore, we investigated ddcfDNA trends in kidney (KT) and liver (LT) recipients over the first 2 years post-transplantation, assessing intra- and inter-individual variability in absolute and relative quantities. Additionally, we analysed urinary ddcfDNA in KT alongside plasma ddcfDNA and other biomarkers.

**Methods:**

Blood and urine from KT and LT were collected longitudinally at regular visits. Mismatched *HLA-DRB1* alleles between donor and recipient were used to determine absolute (ddcfDNA_cpml) and relative (ddcfDNA%) quantities of ddcfDNA with droplet digital PCR.

**Results:**

We found comparable inter- and intra-individual variability in stable KT plasma for both absolute and fractional ddcfDNA. In stable and nonstable LT plasma, intra-individual variability accounted for most of the total variance. Urinary ddcfDNA in KT showed inter-sex variability in fractional quantities.

**Conclusions:**

The large contribution of intra-individual variability suggests including individual ddcfDNA dynamics in addition to fixed cut-offs for the detection of allograft injury. In LT and urinary ddcfDNA in KT, ddcfDNA_cpml should be considered alongside ddcfDNA%, as they seem to directly represent the allograft status.

## 1 Introduction

Donor-derived cell-free DNA (ddcfDNA) has emerged as a non-invasive biomarker for detecting allograft injury in solid organ transplant recipients (1, 2). In kidney and liver recipients, elevated blood ddcfDNA (p-ddcfDNA) has been associated with acute rejection or other allograft dysfunctions (1–4). Despite its benefit in clinical diagnostics (1, 5), many factors contributing to ddcfDNA variability remain unclear. Intra-patient variability in ddcfDNA levels may reflect temporal changes in immunological activity, medical adherence, or graft health, while inter-patient variability could be influenced by graft type, or baseline turnover of ddcfDNA (6, 7). These sources of variability could influence clinical decision-making and suggest a potential benefit of individual dynamics to be taken into consideration in addition to fixed thresholds.

A predictive value of ddcfDNA within 24 h after transplantation correlating with 6-month post-transplantation estimated glomerular filtration rate (eGFR) in kidney recipients was reported by Cucchiari et al. (8). Additionally, in kidney recipients with only low ddcfDNA (<0.5%) measurements after the first month post-transplantation, there was a significant increase in the eGFR from 5 to 12 months after transplantation (9), suggesting some prognostic value of early ddcfDNA quantities. No studies have yet examined ddcfDNA and long-term allograft function in liver recipients.

There is a wide range of different methods available for analysing ddcfDNA (10, 11). Based on previous studies in our group, we utilised a ddPCR approach with *HLA-DRB1* mismatch (12–15). This method requires HLA typing of both recipient and donor in advance, which is usually part of the transplantation process and therefore genotype information is readily available. Another advantage of this analysis method was the necessity for only two assays (donor-specific *HLA-DRB1* allele and a reference gene) per patient, reducing analysis costs. However, if no *HLA-DRB1* mismatch is present between the recipient and donor or if there is a cross-reaction of the assays with other HLA genes, ddcfDNA quantification is not possible with this method. In contrast to sequencing-based methods, ddPCR enables measurement of absolute cfDNA quantities. While most studies focus on fractional ddcfDNA in plasma (p-ddcfDNA%), incorporating absolute ddcfDNA (p-ddcfDNA_cpml) quantities has been shown to improve the prediction of allograft rejection (5, 16). Additionally, there is very limited data published on ddcfDNA in urine (u-ddcfDNA), although initial findings suggested a clinical relevance (17, 18). U-ddcfDNA offers potential advantages such as direct sampling as well as less volume restriction compared to a blood sample. However, u-ddcfDNA also poses technical challenges, due to its increased fragmentation, heterogeneous size distribution, and other factors influencing extraction and detection methods (19, 20). The cfDNA in urine consists of both transrenally filtered cfDNA as well as DNase degraded cfDNA both from the urogenital tract and transrenal cfDNA (21). Therefore, it is likely that u-ddcfDNA in kidney recipients originates primarily from direct shedding rather than filtration through the kidneys. This release of ddcfDNA directly into the urine could give additional insights into the allograft health status.

For a better understanding of ddcfDNA dynamics, this study aimed to determine and compare intra- and inter-individual variability in stable and nonstable kidney and liver recipients for absolute and relative ddcfDNA quantities. Furthermore, we investigated ddcfDNA early after transplantation as a potential prognostic factor for allograft function within the first year after allograft implantation. Our study further aimed to examine u-ddcfDNA in kidney recipients and assess its correlation with corresponding p-ddcfDNA measurements.

## 2 Materials and methods

### 2.1 Study cohort

Kidney (KT) and liver (LT) transplant recipients, as well as healthy control subjects, were recruited at the Inselspital, Bern University Hospital, Switzerland. Inclusion criteria were predefined as patients aged >18 years, feasible blood sampling, and no current pregnancy, as well as kidney or liver transplantation for KT and LT, respectively. Exclusion criteria for healthy control subjects consisted of <18 years of age, previous organ transplantation, blood transfusion, administration of plasma-derived products, current or recent (<3 months) pregnancy, or current known malignancies. The study was performed in accordance with the ethics approval granted by the Ethics Committee of the Canton of Bern, Switzerland (2020-00953).

### 2.2 Patient categorisation

KT and LT were classified as stable, nonstable or early post-transplant based on the criteria summarised in Table 1. For the stable group, we aimed to include solely patients without any signs of ongoing allograft damage, stable allograft function as determined by laboratory parameters, and no signs of inflammation, which could result in an increase of recipient cfDNA. The sample timepoints for the stable and nonstable groups were chosen to exclude any early post-transplantation kinetics while leaving sufficient time in-between samples. Furthermore, blood transfusions were included in the categorisation to either take into account (early post-transplant group) or leave sufficient time to remove the influence of additional cfDNA (22).

**Table 1:** Criteria for patient categorisation.

Patient category	Grouping criteria
Stable KT	- All measurements or interventions after 30 days post-transplantation were taken into account. - No allograft biopsy up to 3 months after the last sample collection. - The following criteria were considered from 30 days after transplantation up to 30 days after the last sample collection: - No dialysis, no prominent infection (coefficient of variation of leucocytes in blood <1), no chronic kidney disease stage 4 or 5, maximum of one eGFR measurement <30 mL/min per 1.73 m^2^. No transplants other than kidney. - Three plasma samples available with the earliest 30 days post-transplantation, at least 30 days apart, and the first and third sample at least 6 months apart. - No blood transfusions 24 h prior to sample collection.
Stable LT	- All measurements or interventions after 30 days post-transplantation were taken into account. - No allograft biopsy up to 3 months after the last sample was collected. - The following criteria were considered up to 30 days after the last sample collection: - No prominent infection (coefficient of variation of leucocytes in blood <1), maximum of one ALAT and/or one ASAT measurement above normal range (>35 U/L). No transplants other than liver. - Three plasma samples available with the earliest 30 days post-transplantation, at least 30 days apart, and the first and third sample at least 6 months apart. - No blood transfusions 24 h prior to sample collection.
Nonstable KT and LT	- Not categorised as stable. - Patients with a for-cause allograft biopsy up to 90 days after a sample collection were included, regardless of the biopsy result. - KT: Decline in eGFR of −1 mL/min per year or more. - LT: Multiple elevated ALAT measurements during the study period. - No transplants other than kidney and liver, respectively. - Three plasma samples available with the earliest 30 days post-transplantation, each at least 30 days apart, and the first and third sample at least 6 months apart. - No blood transfusions 24 h prior to sample collection.
Early post-transplant KT and LT	- No transplants other than kidney and liver. - Plasma samples available either within 48 h post-transplantation and a second sample at least 40 h after the first, or 7 days (±3 days) post-transplantation.

### 2.3 Sample collection and processing

Patient samples were collected during hospital stay directly after transplantation or at regular follow-up clinic visits. Sample collection encompassed 15 mL blood in Sarstedt S-Monovette® 7.5 mL K3E (Sarstedt, Nümbrecht, Germany), one 8.5 mL urine Sarstedt Monovette® (Sarstedt, Nümbrecht, Germany), and up to 50 mL urine in 50 mL DNA LoBind® tubes (Eppendorf SE, Hamburg, Germany) containing 2.5 mL Streck® Urine Preserve (Streck Inc., La Vista, NE, USA). Healthy subjects gave up to 30 mL of blood in Sarstedt S-Monovette® 7.5 mL K3E, urine in one 8.5 mL Sarstedt Monovette®, as well as up to 100 mL urine in 50 mL DNA LoBind® tubes with 2.5 mL Streck® Urine Preserve added per 50 mL tube. Details of the sample processing and cfDNA extraction are provided in the Supplementary Material.

### 2.4 HLA-ddPCR for ddcfDNA quantification

Quantification of ddcfDNA for all samples was performed using droplet digital PCR and the HLA-ddPCR method. In this method, mismatched *HLA-DRB1* alleles between donor and recipient or the *SRY* gene (for male donors in female recipients) were used for absolute donor allele quantification as described by Kueng et al. (12). This method was shown to correlate strongly with a high-throughput sequencing method for the analysis of ddcfDNA% (12). Total cfDNA was quantified using the autologous reference gene *RPP30* assay as previously published (20). All HLA-ddPCR measurements (i.e., *HLA-DRB1*, *SRY*, and *RPP30* measurements) were performed as described in Kueng et al. (12) in triplicates without correction for amplicon length. DdcfDNA quantities were calculated as follows:

$$\begin{array}{r} \mathrm{Absolute}\;\;\mathrm{ddcfDNA}\;[\mathrm{cp}/\mathrm{mL}]=\\ \frac{\mathrm{Total}\;\mathrm{ddcfDNA}\;[\mathrm{cp}]\;\;\mathrm{in}\;\;\mathrm{cfDNA}\;\mathrm{eluate}}{\mathrm{Input}\;\;\mathrm{volume}\;\mathrm{of}\;\;\mathrm{plasma}\;\;\mathrm{or}\;\;\mathrm{urine}\;[\mathrm{mL}]} \end{array}$$

$$\begin{array}{r} \mathrm{Absolute}\;\mathrm{ddcfDNA}\;\mathrm{UCr}\;\mathrm{adj}.\;[\mathrm{cp}/\mu \mathrm{mol}]=\\ \frac{\mathrm{Urinary}\;\mathrm{absolute}\;\;\mathrm{ddcfDNA}\;[\mathrm{cp}/\mathrm{mL}]\;}{\left(\mathrm{UCr}\;[\mu \mathrm{mol}/L]\;*\;1,000\right)} \end{array}$$

$$\mathrm{Fractional}\;\mathrm{ddcfDNA}\text{\%}\;=\frac{\mathrm{Total}\;\mathrm{ddcfDNA}\;[\mathrm{cp}]}{\mathrm{Total}\;\mathrm{cfDNA}\;[\mathrm{cp}]}*100$$

For the ddcfDNA_cpml, the quantity of homozygous copies were used. Absolute ddcfDNA quantities in urine (u-ddcfDNA_cpml) refer to UCr adjusted ddcfDNA quantities throughout this manuscript.

### 2.5 Statistical analysis

All statistical analyses were performed using R v4.1.2. Linear mixed-effects models were calculated with the *lme4* (23) v1.1-32 and *lmerTest* v3.1-3 packages. Multiple samples per individual were modelled as random intercept in all linear mixed-effects models. For models analysing early post-transplantation data, the time since transplantation was added as a fixed effect. In the generalised linear mixed-effects models performed in this study the inter-individual variability was accounted for. Variance components were calculated using the analysis of variance (ANOVA) from the *VCA* v1.4.5 package. To compare ANOVA results from different measurements, the ddcfDNA quantities were normalised by dividing each value by the group mean (any deviation from using the normalised values is explicitly stated in the results section). The *ggplot2* v3.4.2 package was used to create all figures. Pearson's method was used for parametric correlations and Kendall's method for non-parametric correlations. A *p*-value of <0.05 was considered statistically significant. The standard error is abbreviated with SE.

## 3 Results

### 3.1 Patient characteristics

The demographics of the patients included in the analyses, are described in Table 2. Patients were transplanted between September 2020 and November 2023. For the early post-transplantation (post-TPL) cohort the samples included were collected within the first 10 days with a median of 2.6 days post-TPL (*n* = 25 KT, *n* = 22 LT). The longitudinal stable cohort (*n* = 19 KT, *n* = 13 LT) consisted of samples 30–558 days post-TPL with three samples selected per patient (first: median 48 days post-TPL; second: median 169 days post-TPL; third: median 369 days post-TPL). In the nonstable longitudinal cohort (*n* = 17 KT, *n* = 14 LT) the samples were collected 30–517 days post-TPL (first timepoint: median 54 days post-TPL; second timepoint: median 174 days post-TPL; third timepoint: median 359 days post-TPL).

**Table 2:** Demographics of recipients for each patient category.

Recipient characteristics	Early		Stable		Nonstable	
	KT (*n* = 25)	LT (*n* = 22)	KT (*n* = 19)	LT (*n* = 13)	KT (*n* = 17)	LT (*n* = 14)
Sex, *n* (%)						
Female	10 (40.0%)	7 (31.8%)	11 (57.9%)	3 (23.1%)	7 (41.2%)	4 (28.6%)
Male	15 (60.0%)	15 (68.2%)	8 (42.1%)	10 (76.9%)	10 (58.8%)	10 (71.4%)
Age (years), median (IQR)	52.8 (19.4)	59.5 (11.7)	51.4 (16.3)	58.0 (13.6)	57.3 (16.0)	57.3 (10.6)
Total ischemia time (min.), median (IQR)	416 (174)	380 (91.2)	397 (285)	382 (90)	447 (223)	370 (106)
Donor type, *n* (%)						
LD	5 (20.0%)	0 (0.0%)	7 (36.8%)	0 (0.0%)	2 (11.8%)	0 (0.0%)
DBD	17 (68.0%)	14 (63.6%)	7 (36.8%)	11 (84.6%)	12 (70.6%)	9 (64.3%)
DCD	3 (12.0%)	8 (36.4%)	5 (26.3%)	2 (15.4%)	3 (17.6%)	5 (35.7%)
Indication for transplantation, *n* (%)						
ADPKD	4 (16.0%)		3 (15.8%)		5 (29.4%)	1 (7.1%)
Diabetes/Hypertension/Vascular aetiology	8 (32.0%)		4 (21.1%)		4 (23.5%)	
Glomerulosclerosis/-nephritis	10 (40.0%)		7 (36.8%)		4 (23.5%)	
Alcohol-related liver disease		5 (22.7%)		1 (7.7%)		3 (21.4%)
Autoimmune		4 (18.2%)		2 (15.4%)		3 (21.4%)
HCC		5 (22.7%)		4 (30.8%)		3 (21.4%)
Metabolic liver disease		4 (18.2%)		1 (7.7%)		2 (14.3%)
Virus-induced liver cirrhosis		2 (9.1%)		2 (15.4%)		
Other (e.g., reflux nephropathy, secondary sclerosing cholangitis)	3 (12.0%)	2 (9.1%)	5 (26.3%)	3 (23.1%)	4 (23.5%)	2 (14.3%)
Retransplant, *n* (%)						
Yes	4 (16.0%)	2 (9.1%)	4 (21.1%)	1 (7.7%)	3 (17.6%)	1 (7.1%)
No	21 (84.0%)	20 (90.9%)	15 (78.9%)	12 (92.3%)	14 (82.4%)	13 (92.9%)

LD, living donation; DCD, donation after circulatory death; DBD, donation after brain death; ADPKD, autosomal dominant polycystic kidney disease; HCC, hepatocellular carcinoma. Total ischemia time included warm (as well as functional warm) and cold ischemia time.

### 3.2 ddcfDNA early post-transplantation

**Figure 1:**
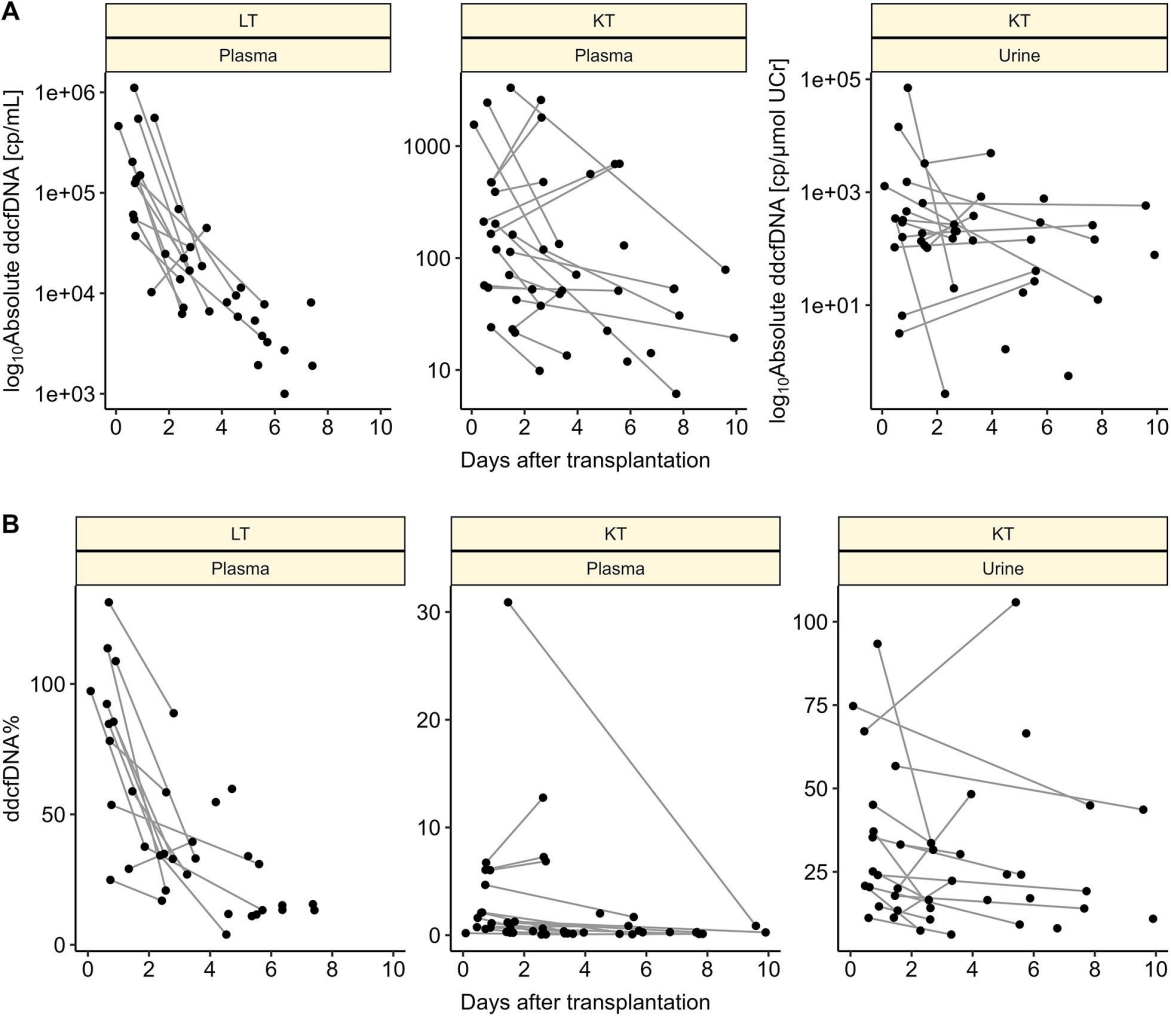
Early post-transplantation ddcfDNA dynamics. Patients with multiple samples are connected by lines. The upper figures **(A)** are representing the absolute ddcfDNA quantities and the lower panels **(B)** the fractional. *Y*-axis values of absolute ddcfDNA were plotted on a log_10_-scale. KT plasma (*n* = 25 patients, *n* = 45 samples), KT urine (*n* = 24 patients, *n* = 41 samples), and LT plasma (*n* = 22 patients, *n* = 36 samples).

P-ddcfDNA within the first 10 days post-transplantation showed a general declining trend for KT (*n* = 25 patients) and a strong decline in LT (*n* = 22 patients) both for fractional and absolute quantities as shown in Figure 1. Conversely, urinary fractional ddcfDNA (u-ddcfDNA%) of KT (*n* = 24 patients) showed no clear dynamic, whereas absolute urinary ddcfDNA (u-ddcfDNA_cpml) quantities declined over the first few days post-transplantation (Figure 1). No statistical testing was performed on these measurements due to the small sample size with multiple samples in the required time frame. In LT the type of donor did not show distinct p-ddcfDNA quantities to be able to differentiate DCD (donation after circulatory death) from DBD (donation after brain death) (Supplementary Figure S1). Similarly, in KT there was no visible distinction between donor types, although, the distribution between donor types was less balanced compared to LT (Supplementary Figure S1). No significant correlation was found between the ischemia time and ddcfDNA (absolute and fractional of KT and LT) within 48 h nor 7 days (±3 days) post-transplantation (Supplementary Table S1). Neither ddcfDNA_cpml nor ddcfDNA% at 7 days (±3 days) in KT nor LT significantly predicted of allograft function (eGFR and ALAT, respectively) at 6 or 12 months post-transplantation (Supplementary Table S2).

### 3.3 Impact of the time post-transplantation on ddcfDNA quantities

Elucidating the effect of time after transplantation within the first 2 years may aid in interpreting ddcfDNA throughout the allograft life. Therefore, we studied the impact of the time post-TPL (days) on ddcfDNA quantities using a linear mixed-effects model adjusting for inter-patient variability in stable and nonstable KT (*n* = 19 stable and *N* = 17 nonstable patients) and LT (*n* = 13 stable and *n* = 14 nonstable patients). A significant positive correlation was found between p-ddcfDNA% and the time post-transplantation in stable LT, as well as p-ddcfDNA% in nonstable KT (Supplementary Table S3). Other ddcfDNA quantities in either plasma or urine were not significantly influenced by the time since transplantation within the first 2 years post-transplantation. The detailed results of all these models are provided in Supplementary Table S3.

### 3.4 Comparison of plasma and urinary ddcfDNA in KT

Investigating potential relationships between p-ddcfDNA and u-ddcfDNA quantities in KT may provide further insights into the excretion of ddcfDNA into other body fluids. After accounting for repeated measures within individuals the linear mixed-effects model revealed a significant effect of p-ddcfDNA% on u-ddcfDNA% only in stable KT [*β* = 37.0, SE = 12.2, *t*_(39)_ = 3.05, *p* = 0.004]. The same model for ddcfDNA_cpml in stable [*β* = 0.89, SE = 0.51, *t*_(53)_ = 1.73, *p* = 0.09] and both absolute and fractional in nonstable [*β* = 0.19, SE = 0.11, *t*_(39)_ = 1.74, *p* = 0.09 resp. *β* = −12.7, SE = 12.5, *t*_(34)_ = −1.02, *p* = 0.32] KT showed no significant effect of plasma on urine. Similarly, in the early post-transplant group no significant correlation was found of plasma on urine for both ddcfDNA_cpml [*β* = −0.37, SE = 2.37, *t*_(38)_ = −0.16, *p* = 0.87] and ddcfDNA% [*β* = 0.46, SE = 0.66, *t*_(33)_ = 0.70, *p* = 0.49]. The data of the comparisons between p-ddcfDNA and u-ddcfDNA are shown in Supplementary Figure S2.

To examine the effect of glomerular filtration rate (eGFR) on u-ddcfDNA in KT, as the rate at which plasma cfDNA might be excreted into the urine, we investigated their potential correlation. However, no significant correlation between eGFR and u-ddcfDNA in either stable or nonstable group was found, when accounting for multiple samples per patient. Similarly, the early post-transplantation group showed no significant correlation between u-ddcfDNA and eGFR when accounting for multiple samples and the time after transplantation (Data not shown).

### 3.5 Variance components for longitudinal ddcfDNA

For the analysis of variance, stable and nonstable patient groups with three samples per patient were analysed for inter-sex, inter-individual, as well as intra-individual variability of ddcfDNA quantities. Nonstable KT patients consisted of 12 cases of for-cause allograft biopsies, including 4 biopsy-proven rejections, as well as 5 cases with low eGFR. The nonstable LT included 3 cases of biopsy-proven rejections and 11 patients with multiple elevated ALAT levels.

**Figure 2:**
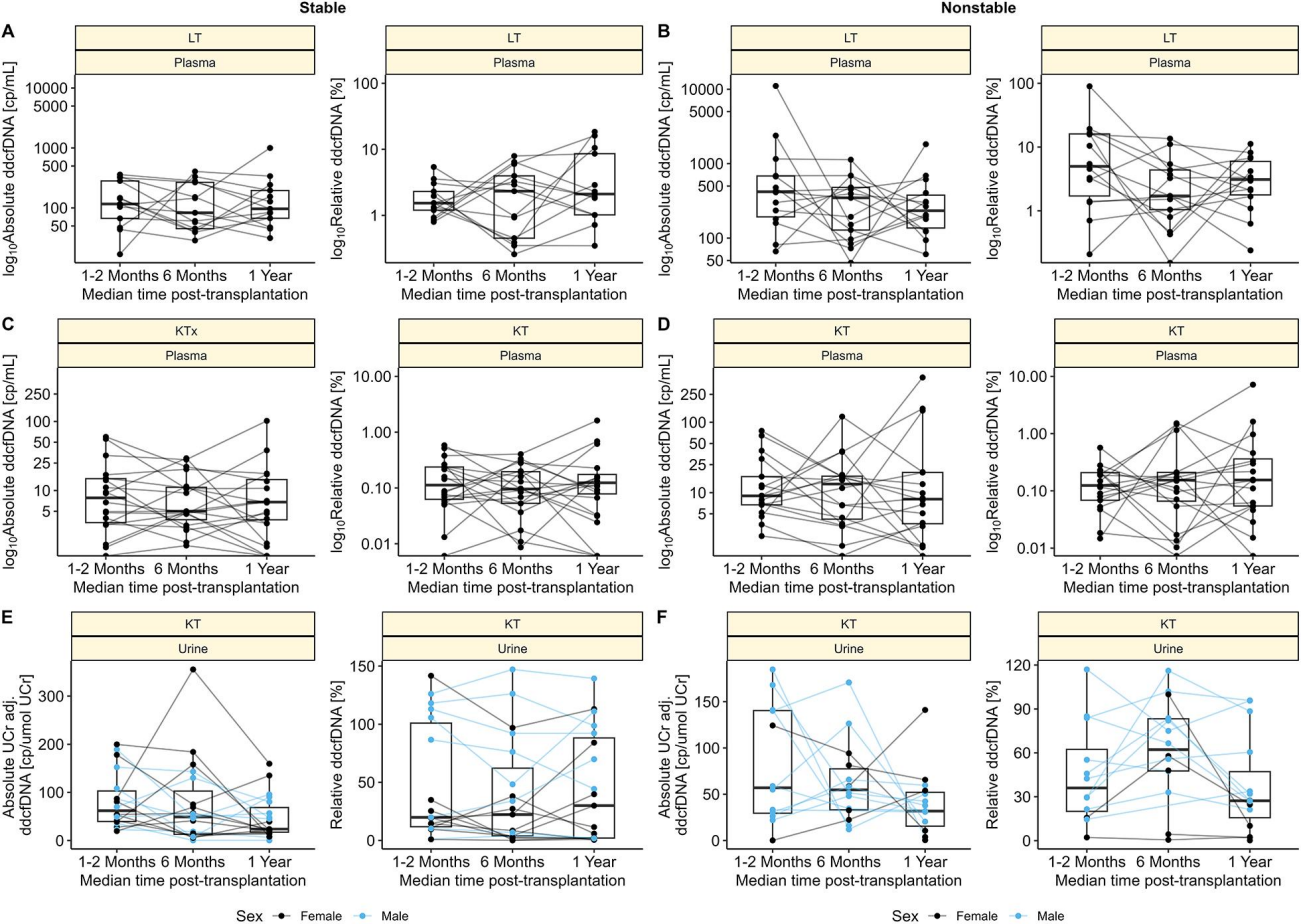
ddcfDNA quantities of stable and nonstable patients. Absolute and relative ddcfDNA is shown for both KT and LT in plasma and urine, where applicable. The urine data is further stratified by the recipient sex. The lines connect measurements from the same individual. For better readability, measurements were grouped according to the time after transplantation. This grouping was used only for this figure and was not used in any of the statistical analyses. The figures **(A)**, **(C)**, and **(E)** show data from stable patients (KT: *n* = 19 patients, *n* = 57 plasma samples, *n* = 56 urine samples; LT: *n* = 13 patients, *n* = 39 plasma samples) and the panels **(B)**, **(D)**, and **(F)** from nonstable categorised patients (KT: *n* = 17 patients, *n* = 51 plasma samples, *n* = 41 urine samples; LT: *n* = 14 patients, *n* = 42 plasma samples). All log_10_ transformed *y*-axes for better data presentation are marked accordingly. A figure without grouping of samples, showing the actual time since transplantation on the *x*-axis is available in the Supplementary Figure S3.

The overall median ddcfDNA% was higher in nonstable vs. stable KT [plasma: 0.149 (IQR = 0.161) vs. 0.096% (IQR = 0.163); urine: 42.4% (IQR = 60.6) vs. 21.1% (IQR = 83.9)] as well as for LT [3.26% (IQR = 5.48) vs. 1.9% (IQR = 2.46)]. Similarly, the median ddcfDNA_cpml was lower stable vs. nonstable group for KT [plasma: 5.01 cp/mL (IQR = 10.4) to 8.36 cp/mL (IQR = 13.4); urine: 48.3 cp/μmol UCr (IQR = 67.1) to 48.0 cp/μmol UCr (IQR = 39.6)] and for plasma of LT from 111 cp/mL (IQR = 202) to 300 cp/mL (IQR = 447). Patient-level dynamics of stable and nonstable recipients are shown in Figure 2 and Supplementary Figure S3.

Individual patient dynamics for stable and nonstable LT are shown in Supplementary Figures S4, S5, revealing that ddcfDNA_cpml and ddcfDNA% followed similar dynamics in most cases. In stable and nonstable KT, some patients had similar trends for ddcfDNA_cpml and ddcfDNA% in plasma and urine alike (Supplementary Figures S6, S7). However, while the ddcfDNA quantities within one body fluid seemed to show similar dynamics, these dynamics differed between sample types.

**Figure 3:**
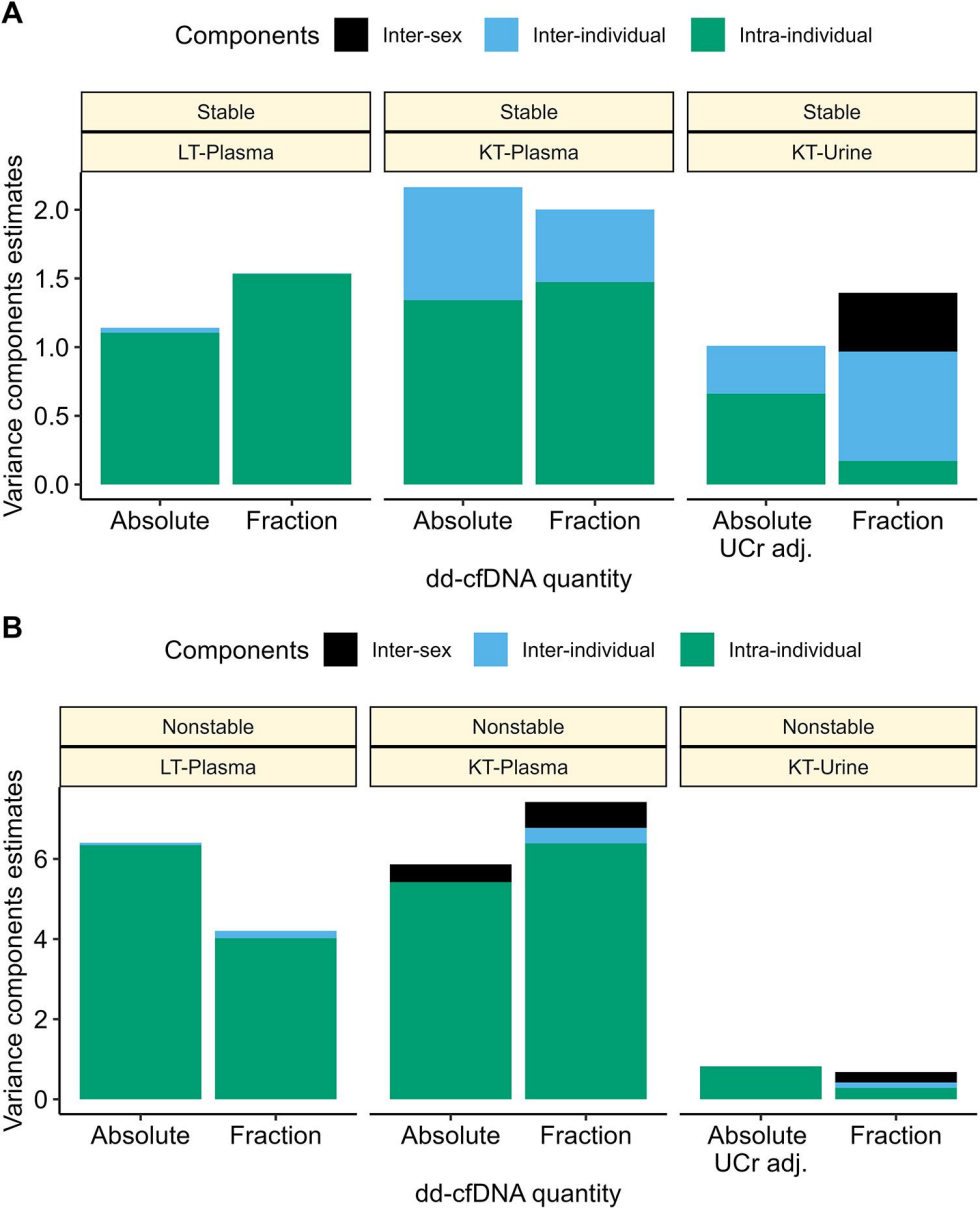
Variance components of ddcfDNA in stable and nonstable patients. The results from stable patients are shown in panel **(A)** and from nonstable patients in panel **(B)** There were *n* = 19 stable KT (*n* = 57 plasma samples, *n* = 56 urine samples) and *n* = 13 stable LT (*n* = 39 plasma samples) included in the models. For the nonstable group, *n* = 17 KT (*n* = 51 plasma samples, *n* = 41 urine samples) and *n* = 14 LT (*n* = 42 plasma samples) were included.

The ANOVA in LT showed the majority of variance attributed to intra-individual variability for both p-ddcfDNA_cpml and p-ddcfDNA% with only a marginal contribution of inter-individual variability and higher overall variability for fractional quantities (Figure 3). P-ddcfDNA in KT showed similar inter- and intra-individual variability for patients categorised as stable, and similar overall variability between p-ddcfDNA_cpml and p-ddcfDNA%. In contrast intra-individual variability increased in nonstable KT for both p-ddcfDNA_cpml and p-ddcfDNA% (shown in Figure 3, Supplementary Figure S8). To investigate whether nonstable KT show stronger fluctuations in ddcfDNA even if ddcfDNA quantities never exceeded current clinical thresholds, we performed an ANOVA of stable and nonstable KT with all measurements either below 1% and 100 cp/mL or 0.5% and 50 cp/mL. We observed similar intra- and inter-individual variability between stable and nonstable patients (Supplementary Figure S9) and thus did not find evidence of increased intra-individual variability in nonstable KT without ddcfDNA measurements exceeding these cut-offs.

Inter-sex variability accounted for part of the inter-individual variability in u-ddcfDNA% of stable KT (Figure 3A), which was mirrored by female patients having generally lower fractions compared to male patients (Figure 2E). In nonstable KT this difference was similarly visible in both the actual measurements (Figure 2F) as well as in the respective inter-sex variability component of the ANOVA (Figure 3B). Interestingly, the amount of ddcfDNA% variance explained by inter-individual variability was greater in urine than in plasma for stable KT.

### 3.6 Correlation with other biomarkers

**Figure 4:**
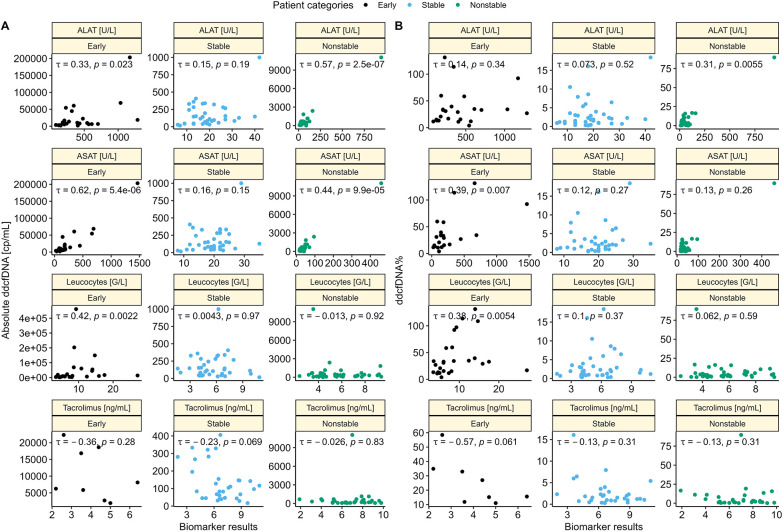
Correlations of biomarkers with ddcfDNA in LT. The correlations were performed separately on each patient category for absolute ddcfDNA quantities (**A**) and fractional ddcfDNA (**B**). All shown correlations were generated using Kendall's tau.

The overall correlation with all samples using Kendall's method was significant for p-ddcfDNA_cpml and p-ddcfDNA% in LT with the liver enzymes ALAT and ASAT (Supplementary Table S4). This correlation was primarily driven by the early and nonstable patient groups, whereas in the stable group, where only one elevated ALAT or ASAT value was allowed, the dynamic range of liver enzymes was narrower. In LT, the leucocyte count in blood correlated significantly with p-ddcfDNA, the largest contribution coming from the early post-transplantation samples (Figure 4). For LT both p-ddcfDNA_cpml and p-ddcfDNA% showed a negative correlation with tacrolimus levels that could not be attributed to a specific patient category. Tacrolimus blood levels correlated positively with u-ddcfDNA_cpml in KT (Supplementary Table S4). In KT, we found significant correlations between p-ddcfDNA and creatinine blood levels as well as its corresponding eGFR (Supplementary Table S4). These correlations were not confined to any single patient group, whereas a correlation between p-ddcfDNA_cpml and CRP was observed in stable KT (Supplementary Figure S10). U-ddcfDNA_cpml in KT correlated significantly with creatinine blood levels, eGFR, leucocyte counts in blood, and CRP when considering all samples, but not within any individual patient group (Supplementary Table S4, Supplementary Figure S11).

## 4 Discussion

In this study, we observed greater intra-individual variability in ddcfDNA among nonstable KT and LT compared to stable recipients within the first 2 years after transplantation. While overall variability was similar for p-ddcfDNA_cpml and p-ddcfDNA% in KT, the difference of intra-individual variability between stable and nonstable recipients was more pronounced in LT for p-ddcfDNA_cpml, indicating an advantage of quantifying absolute ddcfDNA in these patients. Furthermore, u-ddcfDNA appeared largely independent of p-ddcfDNA in KT with a potential correlation only observed for ddcfDNA% in stable KT. Sex-based and pronounced inter-individual differences in u-ddcfDNA%, on the other hand, suggest that u-ddcfDNA_cpml levels may serve as a more robust and recipient-independent urinary biomarker in KT.

### 4.1 Increased intra-individual variability in nonstable patients

The observed shift of almost equal inter- and intra-individual variability in stable KT plasma to predominantly intra-individual variability in nonstable KT patients, together with increased intra-individual variability in nonstable LT, highlights the potential utility of ddcfDNA for personalised allograft monitoring. The benefit of including ddcfDNA_cpml quantities in the evaluation of ddcfDNA has been demonstrated by several studies (5, 16). We found the total variability and its components between p-ddcfDNA_cpml and p-ddcfDNA% in KT to be similar, suggesting that both quantification approaches may provide complementary information.

For KT, early post-transplantation ddcfDNA quantities did not correlate with allograft function at either 6 or 12 months after transplantation, contrary to a published study (8). As this discrepancy might be due to the lower sample size in our study, further investigation of the predictive value of early post-transplantation ddcfDNA quantities is warranted.

### 4.2 Importance of absolute ddcfDNA in liver recipients

In LT the correlations between liver enzymes (ALAT and ASAT) and p-ddcfDNA reflect their common origin due to liver tissue injury. Correlations of p-ddcfDNA_cpml with ALAT and ASAT were stronger, which may indicate confounding of p-ddcfDNA% by variable recipient cfDNA quantities. Similarly, in LT, intra-individual variability was lower for p-ddcfDNA_cpml in stable recipients and increased more markedly in nonstable patients. p-ddcfDNA% has been shown to distinguish allograft rejection as well as subclinical rejection from stable LT (3, 24). Taken together, these findings suggest that in liver recipients p-ddcfDNA_cpml should be quantified and included in the interpretation in conjunction with fractional quantities. Given the minimal contribution of inter-individual variability in both stable and nonstable LT patients, our findings do not indicate substantial inter-individual differences in baseline p-ddcfDNA quantities. Our study does not provide any evidence for an added benefit of p-ddcfDNA monitoring during the first 10 days post-transplantation in liver recipients, given the lack of predictive value of early p-ddcfDNA levels for 1-year allograft function. However, these results may have been confounded by perioperative blood transfusions or a small sample size. The observed correlation of p-ddcfDNA% with time after transplantation may reflect reductions in immunosuppression that affect not only ddcfDNA, but also recipient cfDNA quantities. While a similar trend was reported by Schütz et al. (25) for long-term follow-up of KT, given the possibility of a type I error, this finding requires replication in independent cohorts.

### 4.3 Lack of correlation between urinary and plasma in KT

This study is among the first to describe variability in u-ddcfDNA in KT over the first 2 years after transplantation. In u-ddcfDNA%, inter-individual and inter-sex differences outweighed intra-individual variability. This highlights the importance of considering differences between the sexes as well as other individual-specific factors, which might be due to anatomical reasons, urinary composition, or collection method (26). Although the correction of u-ddcfDNA_cpml with urinary creatinine was shown to reduce the total variability and make the u-ddcfDNA_cpml quantities more comparable (20), it might be useful to further explore u-ddcfDNA% in the context of biomarkers, e.g., leucocytes in urine. Nevertheless, our findings strongly emphasise the importance of including u-ddcfDNA_cpml quantification in any investigations of u-ddcfDNA. The significant effect of p-ddcfDNA% on u-ddcfDNA in stable but not in nonstable KT, could reflect a steady-state during an allograft quiescent period, but requires further investigation to assess reproducibility of this finding. Conversely, the independence of p-ddcfDNA and u-ddcfDNA in nonstable KT might reflect the direct excretion of cfDNA from the allograft into the urine differing from release into plasma, particularly in non-quiescent conditions. Given the limited sample size of this study, further investigations of paired plasma and urine samples should be conducted to further elucidate associations between ddcfDNA quantities in the two body fluids under varying allograft conditions.

During the early post-transplantation phase, u-ddcfDNA showed more constant levels compared to p-ddcfDNA. This effect may be explained by the initial ischemia-reperfusion damage, caused by the limited capacity of peritubular capillaries in kidneys during the reperfusion state to repair ischemic damage during reperfusion (27). We found early p-ddcfDNA_cpml quantities to be elevated almost ten times compared to u-ddcfDNA_cpml levels after 30 days post-transplantation, indicating an extended duration of increased ddcfDNA release into urine compared to p-ddcfDNA. Additionally, u-ddcfDNA might be beneficial in diagnosing BK polyomavirus-associated nephropathy (17) and potentially even TCMR, as the ddcfDNA from tubular cells might be excreted through urine and could lead to earlier increases of u-ddcfDNA compared to plasma. However, in our study we were not able to investigate this due to a low number of patients with either allograft rejections or BK polyomavirus-associated nephropathies.

### 4.4 ddcfDNA as a potential biomarker for immunosuppression monitoring

Correlations between tacrolimus blood levels and ddcfDNA revealed organ-specific patterns that may reflect differences in immunosuppressive response and graft vulnerability. In KT, tacrolimus levels measured in blood on the same day as the ddcfDNA sample collection showed a significant positive correlation with u-ddcfDNA_cpml but not with p-ddcfDNA. This finding might indicate nephrotoxic effects of elevated tacrolimus exposure, resulting in increased renal cell injury and subsequent release of ddcfDNA into urine. In contrast, in LT we found a significant correlation of higher tacrolimus levels corresponding to lower p-ddcfDNA_cpml and p-ddcfDNA%, consistent with effective immunosuppression and reduced allograft injury. Although a similar inverse relationship might be expected in plasma of KT, the absence of this trend may be attributed to the limited number of samples available with paired tacrolimus measurements in this patient group. These observations highlight that further studies of utilising ddcfDNA in regulating immunosuppression through direct mirroring of allograft health may be warranted. The study by Osuchukwu et al. (28) successfully performed ddcfDNA-guided immunosuppression reduction in KT. Based on our findings, future research projects may extend this approach to LT.

### 4.5 Strengths and limitations

Several limitations of our study should be noted, including the small number of patients with complete paired plasma and urine sampling. Similarly, numbers of specific types of allograft dysfunction were too small (4 biopsy-proven KT rejections, 3 biopsy-proven LT rejections) to allow for separate analyses of outcomes such as ABMR, TCMR, and BKVN. Due to no protocol biopsies being performed at the institution of the study, no biopsy-proven stable patients were available. Another limitation is the relatively short follow-up duration of approximately 2 years post-transplantation. Furthermore, given the substantial number of statistical tests performed, there is an increased risk of type I error as no correction for multiple testing was applied. Additionally, future studies of u-ddcfDNA should not only analyse urinary creatinine but also measure leucocytes and potentially additional parameters to investigate their effects on ddcfDNA and cfDNA in urine.

A key strength of this study is its longitudinal design, which made it possible to evaluate intra- and inter-patient variability of a biomarker, which has been proposed to be used with fixed cut-offs for the detection of allograft rejection. This research shows not only that ddcfDNA_cpml quantities are of importance, but also that intra-patient fluctuations should be taken into account in clinical and research settings.

## Data Availability

The original contributions presented in this study are included in the article/Supplementary material, further inquiries can be directed to the corresponding author.
