# Supplementary material for: Variability and longitudinal dynamics of donor-derived cell-free DNA in kidney and liver recipients: a comparison of absolute and relative quantities in plasma and urine
Source: Front Transplant. 2026 Jun 2;5:1790754. doi: 10.3389/frtra.2026.1790754 (PMC13269088; doi:10.3389/frtra.2026.1790754)

Variability and Longitudinal Dynamics of Donor-Derived Cell-Free DNA in Kidney and Liver Recipients: A Comparison of Absolute and Relative Quantities in Plasma and Urine

Authors: Fanny Sandberg, MD (1,2), Nicholas Kueng, MS (1,2), Vanessa Banz, MD, PhD (3), Annalisa Berzigotti, MD, PhD (3), Daniel Sidler, MD, PhD (4), Carlo R. Largiadèr, PhD (2), Ursula Amstutz, PhD (2)

1 = Graduate School for Cellular and biomedical sciences, University of Bern, Switzerland

2 = Department of Clinical Chemistry, Inselspital, Bern University Hospital, University of Bern, Switzerland

3 = Department of Visceral Surgery and Medicine, Inselspital, Bern University Hospital, University of Bern, Switzerland

4 = Department of Nephrology and Hypertension, Inselspital, Bern University Hospital, University of Bern, Switzerland

SUPPLEMENTAL DIGITAL CONTENT

Sample processing details

Blood samples were centrifuged at 2000x g for 15 min. at room temperature (RT), followed by an additional centrifugation of the plasma at 3800x g for 10 min. at RT and collecting the resulting supernatant for freezing at -80 °C in the local biobank until analysis. Urine in 50 mL tubes was centrifuged at 2000x g for 15 min at RT and the supernatant was stored in 50 mL DNA LoBind® tubes at -20 °C until analysis. Urinary creatinine (UCr) was measured from the 8.5 mL urine without preservative on a cobas® c 702 (Roche Diagnostics International Ltd, Rotkreuz, Switzerland) using the Creatinine plus version 2 enzymatic assay. Samples from healthy volunteers were processed and stored in the same manner, except that the samples were not stored in the biobank but in separate freezers at the same temperatures as patient samples.

cfDNA extraction

CfDNA from plasma samples was extracted using the QIAamp Circulating Nucleic Acid kit (Qiagen, Hilden, Germany). The extraction was performed as previously described[14] with the adjustment of spiking 40000 copies of the CEREBIS CER180bp artificial spike-in instead of 20000 copies. The Quick-DNA Urine Kit (Zymo Research, Irvine, CA, USA) was used for cfDNA extraction from urine as previously described[14] with up to 40 mL urine input and 40000 copies CER89bp, and 40000 copies CER180bp spiked before each extraction. The concentration of all extracted samples was measured with Qubit 1X dsDNA HS Assay Kit on the Qubit 4 fluorometer (Thermo Fisher Scientific, Waltham, MA, USA) and cfDNA was stored at -20 °C.

Figure S1: Early post-transplantation dynamics with type of donor. The absolute (A) and fractional (B) ddcfDNA is shown for samples early post-transplantation. The lines connect samples from the same individual. A log10 transformation was applied to the y-axis of absolute ddcfDNA.

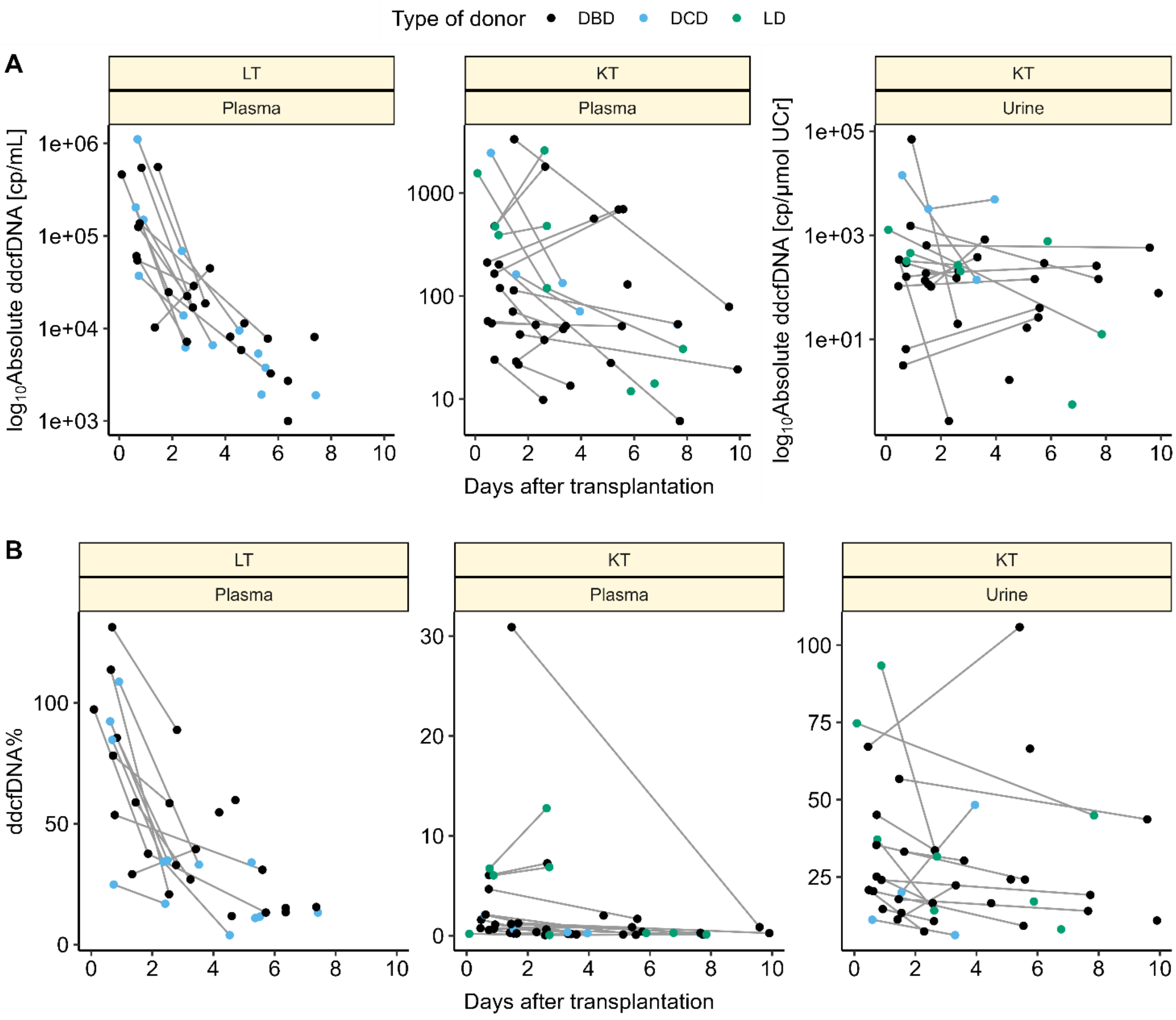

Table S1: Correlation of ischemia time with ddcfDNA at two timepoints post-TPL.

ddcfDNA Time post-TPL	Organ	Fluid	ddcfDNA quantity	Estimate (τ)	p-value	n
<48 hours	Kidney	Plasma	Absolute	0.01	0.940	19
			Fraction	0.12	0.495	19
		Urine	Absolute	0.14	0.433	18
			Fraction	-0.30	0.091	18
	Liver	Plasma	Absolute	0.36	0.116	12
			Fraction	-0.09	0.737	12
7d (+/-3d)	Kidney	Plasma	Absolute	0.03	0.914	14
			Fraction	-0.06	0.830	14
		Urine	Absolute	0.26	0.252	13
			Fraction	0.18	0.435	13
	Liver	Plasma	Absolute	0.051	0.858	13
			Fraction	-0.36	0.1	13

TPL = transplantation

Table S2: Correlation of allograft function at 6 and 12 months post-TPL with ddcfDNA at 7 days post-TPL.

Time post-TPL of Allograft Function	Organ	Fluid	ddcfDNA quantity	Estimate (τ)	p-value	n
6 months (+/- 1 month)	Kidney	Plasma	Absolute	-0.40	0.073	12
			Fraction	-0.28	0.215	12
		Urine	Absolute	-0.31	0.183	11
			Fraction	0.02	0.938	11
	Liver	Plasma	Absolute	0.29	0.178	13
			Fraction	0.18	0.391	13
12 months (+/- 3 months)	Kidney	Plasma	Absolute	-0.21	0.331	14
			Fraction	-0.03	0.914	14
		Urine	Absolute	-0.13	0.590	13
			Fraction	0.21	0.367	13
	Liver	Plasma	Absolute	0.18	0.391	13
			Fraction	0.13	0.540	13

TPL = transplantation

Table S3: Effect of time post-TPL on ddcfDNA

	Fluid	ddcfDNA quantity	Estimate (β)	SE	DF	t-value	p-value
**KT stable**	Plasma	Absolute	-0.003	0.014	31.51	-0.254	0.800
	Plasma	Fraction	9.9E-5	2.1E-4	37.68	0.465	0.645
	Urine	Absolute UCr adj.	-0.104	0.052	36.91	-1.986	0.055
	Urine	Fraction	-0.004	0.018	36.14	-0.216	0.831
**LT stable**	Plasma	Absolute	0.049	0.184	29.51	0.266	0.792
	Plasma	Fraction	0.010	0.004	28.57	2.679	0.012*
**KT nonstable**	Plasma	Absolute	0.111	0.071	36.32	1.564	0.126
	Plasma	Fraction	0.002	0.001	35.44	2.038	0.049*
	Urine	Absolute UCr adj.	-0.111	0.058	39.00	-1.912	0.063
	Urine	Fraction	-0.019	0.032	27.35	-0.601	0.553
**LT nonstable**	Plasma	Absolute	-1.505	1.848	32.44	-0.815	0.421
	Plasma	Fraction	-0.018	0.015	31.69	-1.194	0.241

*The results from the linear mixed-effects model of time after transplantation on ddcfDNA quantities for each of the represented subgroups with multiple samples per patient modelled as random intercept. *Values with p-level <0.05. TPL = transplantation.*

Figure S2: Absolute and fractional ddcfDNA of KT plasma vs. urine. To illustrate the correlation between plasma and urine ddcfDNA in each patient category with the lines connecting samples from the same patient. The axis of the early post-transplantation group were log10-transformed.

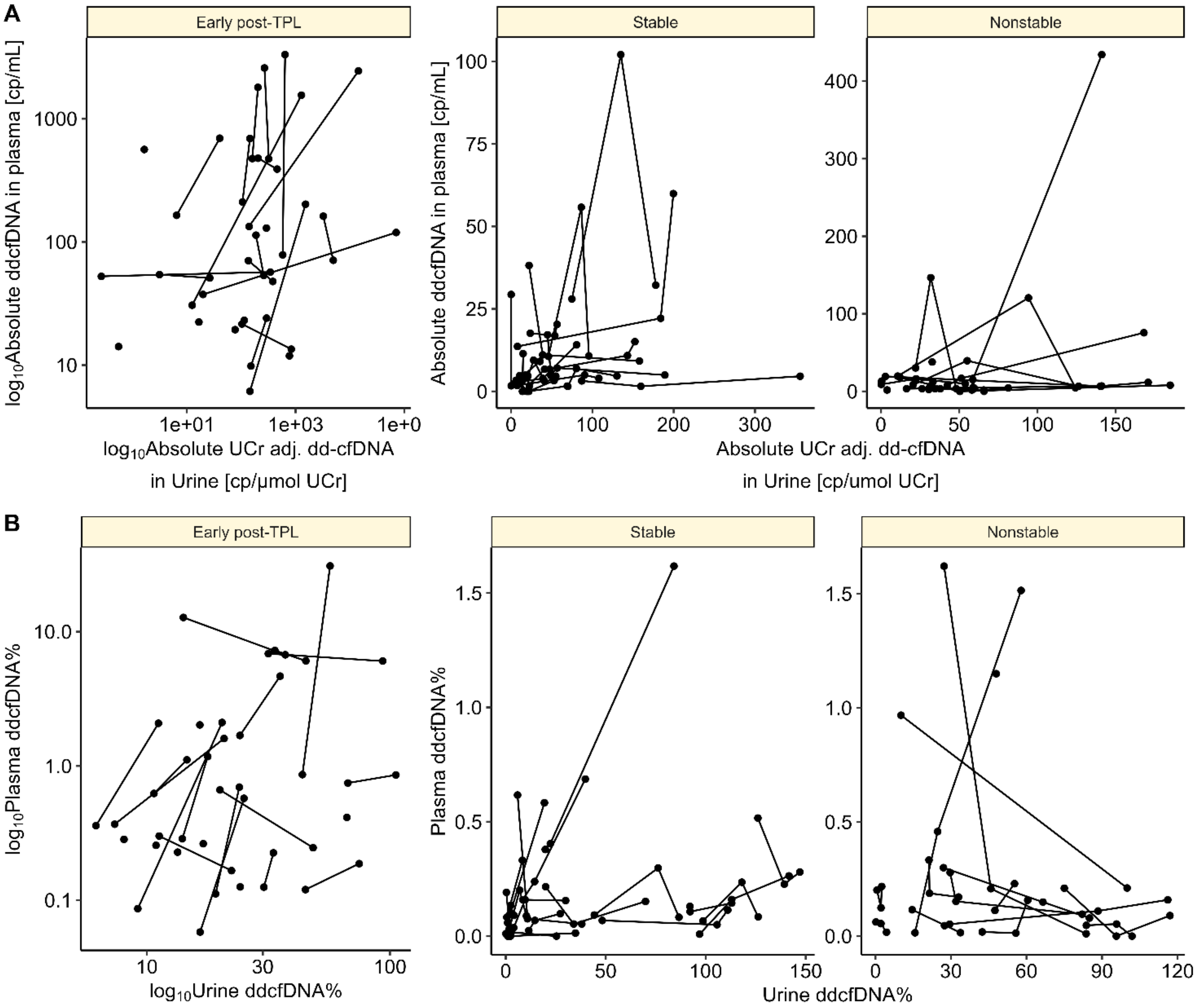

Figure S3: ddcfDNA quantities of stable and nonstable patients. Absolute and relative ddcfDNA is shown for both KT and LT in plasma and urine, where applicable. The urine data is further stratified by the recipient sex. The lines connect measurements from the same individual. The figures (A), (C), and (E) show data from stable patients (KT: n(patients)=19, n(plasma samples)=57, n(urine samples)=56; LT: n(patients)=13, n(plasma samples)=39) and the panels (B), (D), and (F) from nonstable categorised patients (KT: n(patients)=17, n(plasma samples)=51, n(urine samples)=41; LT: n(patients)=14, n(plasma samples)=42). All log10 transformed y-axes for better data presentation are marked accordingly.

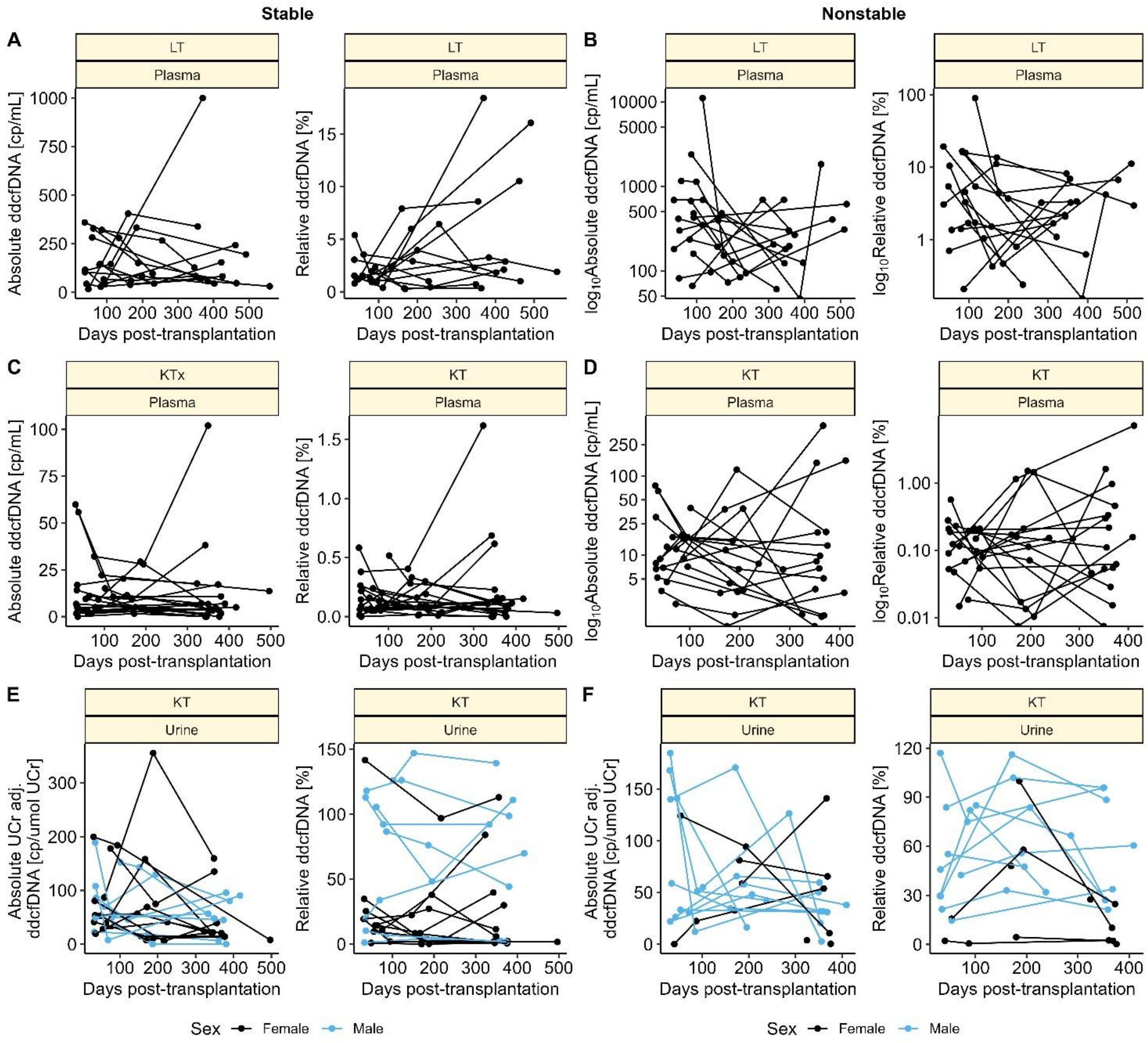

Figure S4: Individual ddcfDNA dynamics of stable LT. All stable LT patients are shown with their individual changes in absolute and fractional ddcfDNA on the same timeline as for ALAT changes.

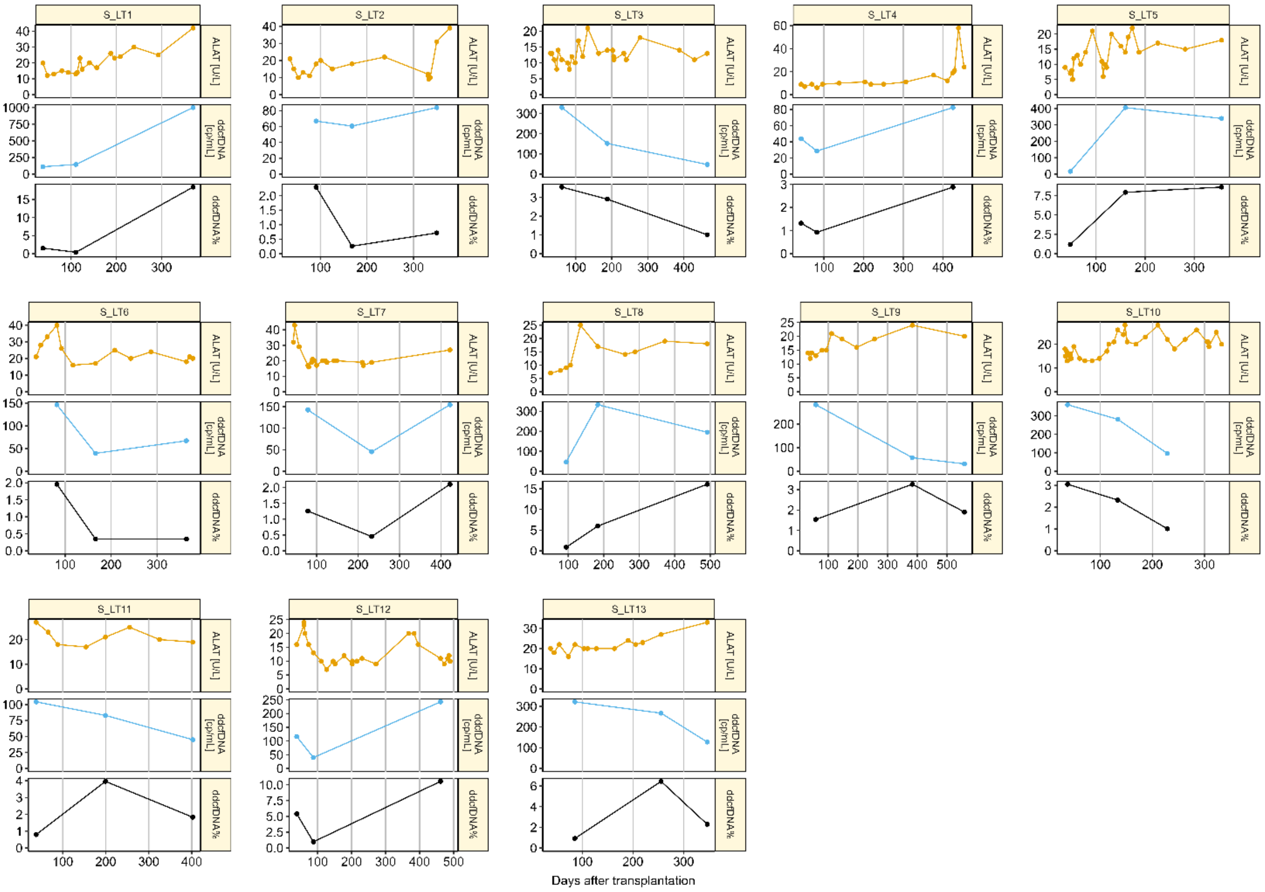

Figure S5:Individual ddcfDNA dynamics of nonstable LT. The absolute and fractional ddcfDNA in LT is shown for each nonstable categorised patient in relation to the liver enzyme ALAT in the top panel.

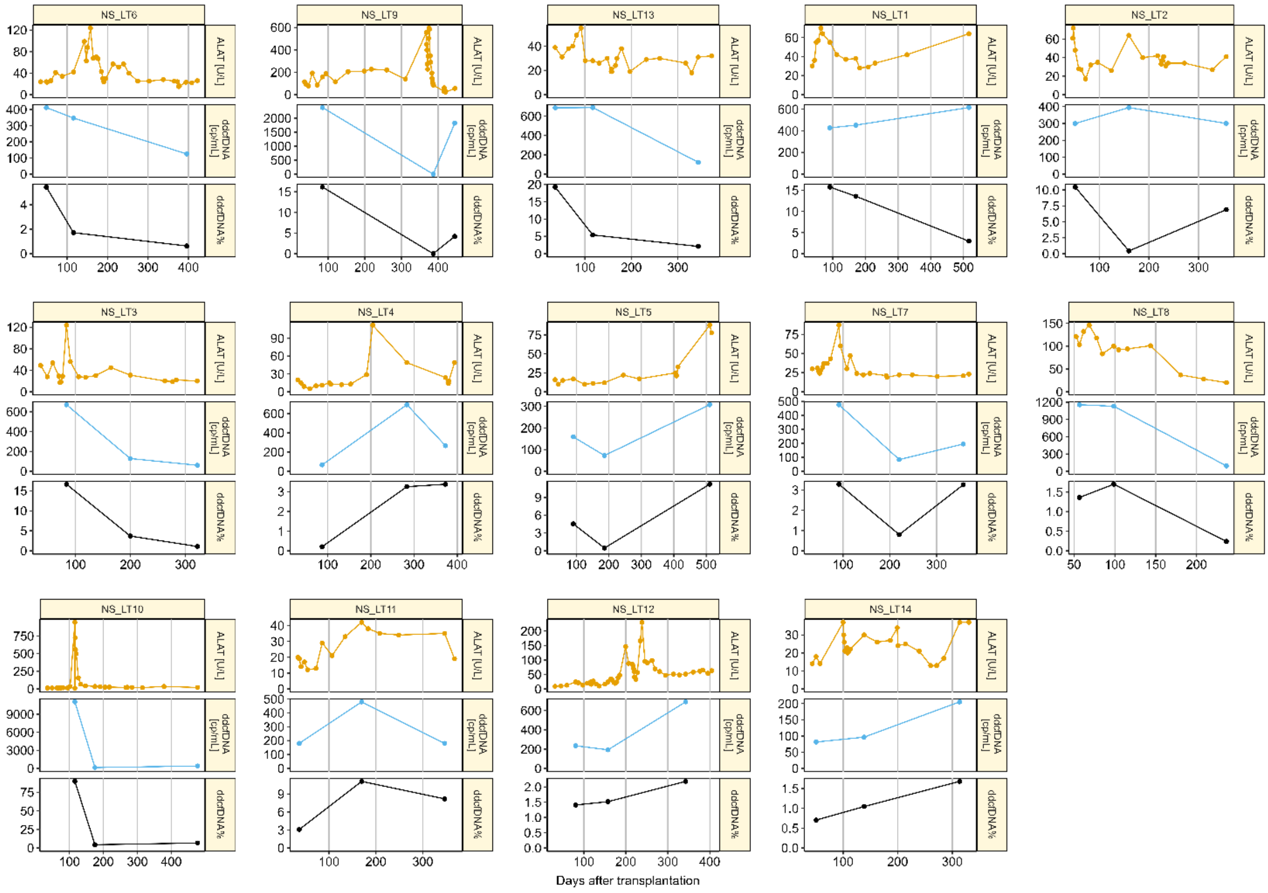

Figure S6: Individual plasma and urine ddcfDNA dynamics for stable KT. The eGFR, absolute and fractional ddcfDNA are shown for each stable KT separately. Absolute ddcfDNA in plasma is depicted in the second panel from the top with fractional quantities from plasma shown in the panel just below. The bottom two panels show the results from urine ddcfDNA.

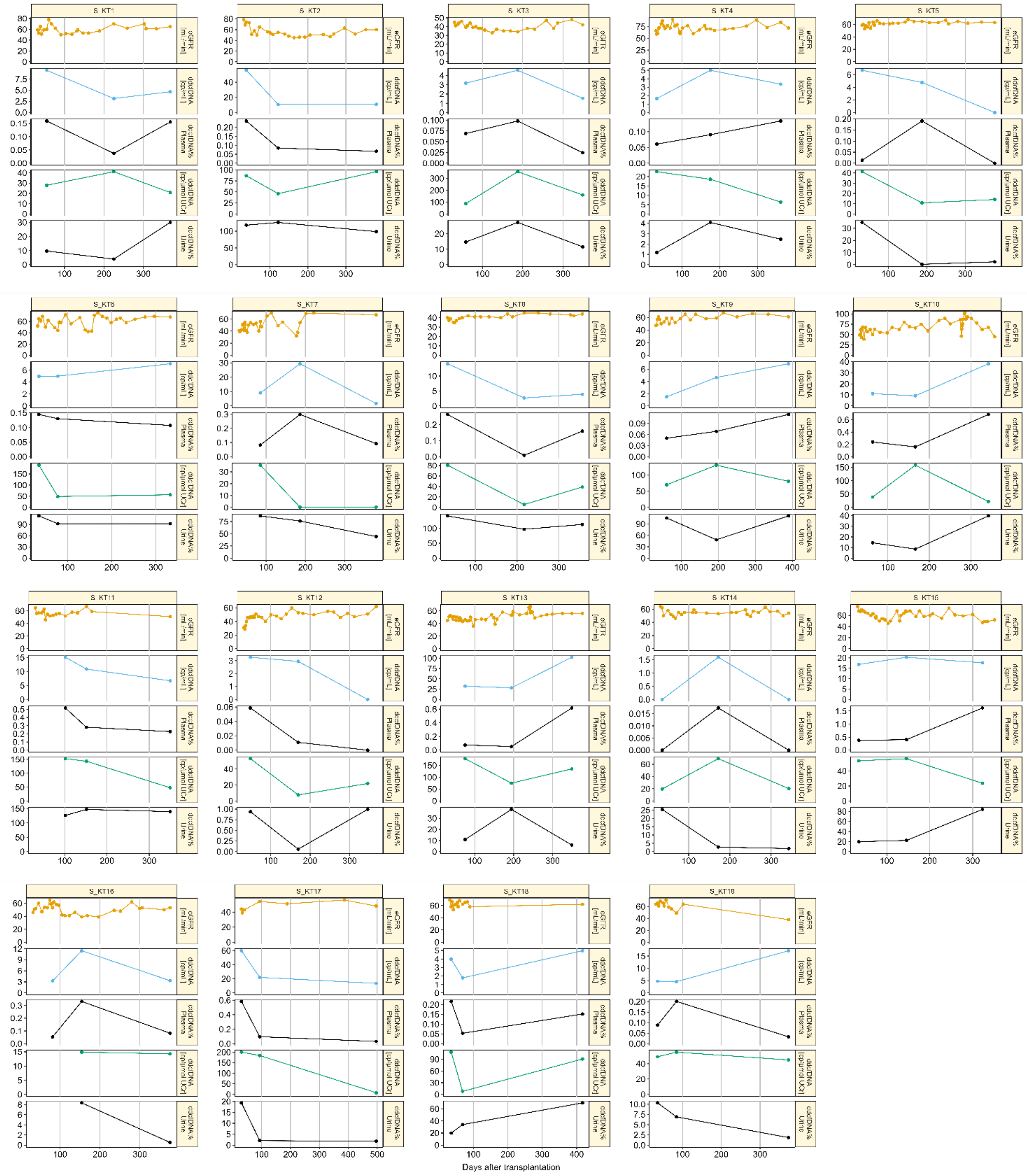

Figure S7: Individual plasma and urine ddcfDNA dynamics in nonstable KT. Each nonstable KT patient is depicted with eGFR in the top panel, absolute and fractional ddcfDNA in plasma in the two below, and ddcfDNA in urine in the bottom two panels.

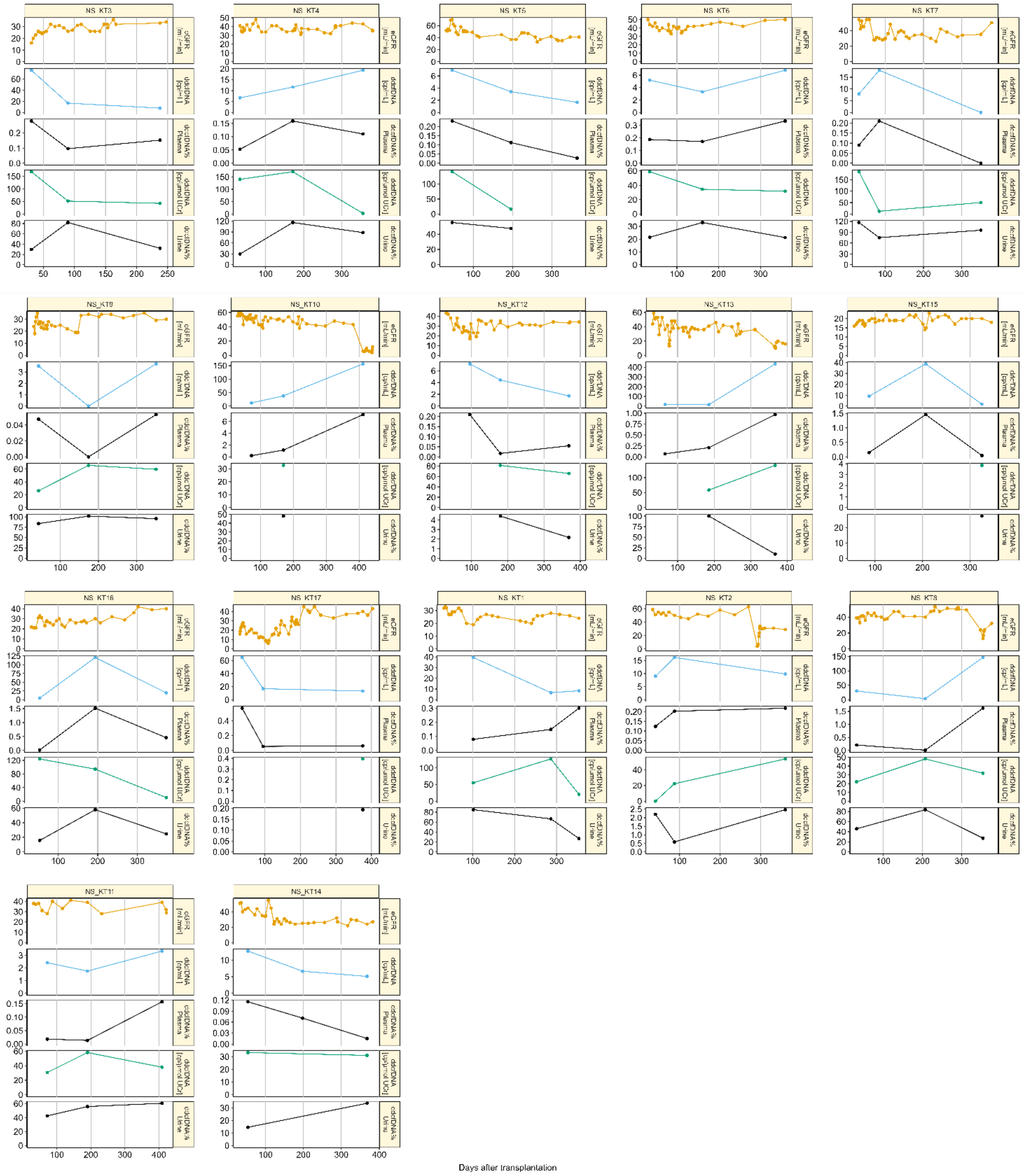

Figure S8: Comparison of ANOVA results from stable and nonstable patients. The panels are grouped to better compare stable and nonstable variance components for each ddcfDNA category.

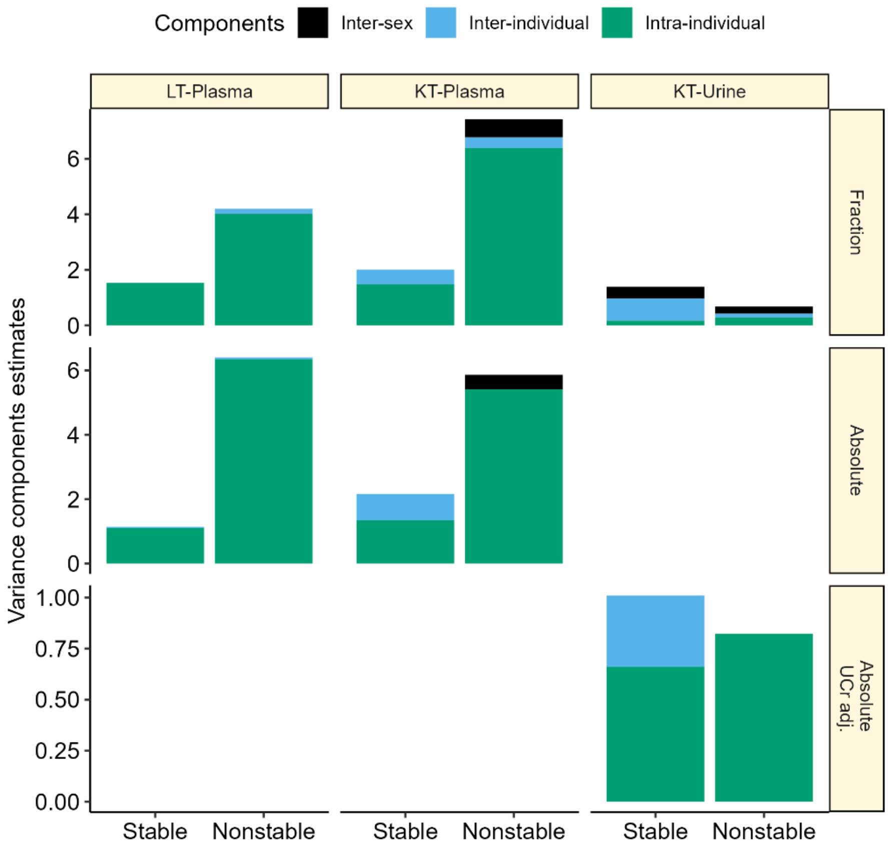

Figure S9: Variance components in plasma of stable KT with upper limits. For the “1% & 100 cp/mL” panel only patients with all three samples below ddcfDNA% of 1% and absolute of 100 cp/mL were included. Similarly, for the “0.5% & 50 cp/mL” analysis.

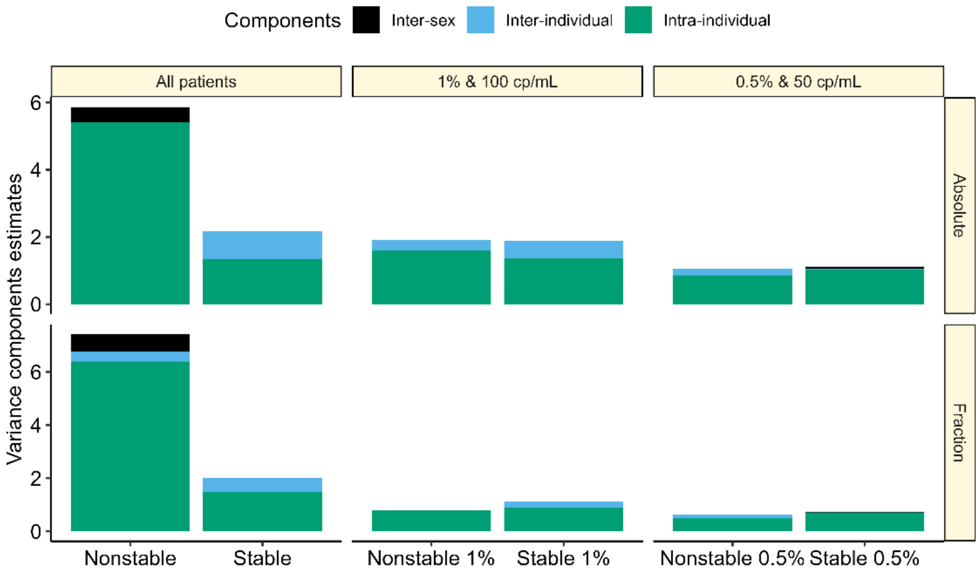

Table S4: Correlation of ddcfDNA with biomarkers

	Fluid	Variable1	ddcfDNA variable	cor (τ)	p-value		n
**KT**	Plasma	Creatinine in blood	Absolute	0.30	9.68E-08	*	145
			Fraction	0.20	4.54E-04	*	145
		CRP	Absolute	0.32	9.85E-06	*	102
			Fraction	0.09	2.18E-01		102
		eGFR	Absolute	-0.29	1.92E-07	*	145
			Fraction	-0.17	2.38E-03	*	145
		Leucocytes in blood	Absolute	0.11	5.46E-02		143
			Fraction	0.11	4.42E-02	*	143
		Tacrolimus level in blood	Absolute	0.16	1.82E-01		34
			Fraction	0.03	8.12E-01		34
	Urine	Creatinine in blood	Absolute UCr adj.	0.19	1.75E-03	*	131
			Fraction	0.10	1.08E-01		131
		CRP	Absolute UCr adj.	0.23	2.25E-03	*	95
			Fraction	0.08	2.89E-01		95
		eGFR	Absolute UCr adj.	-0.17	3.29E-03	*	131
			Fraction	0.02	7.79E-01		131
		Leucocytes in blood	Absolute UCr adj.	0.13	3.25E-02	*	129
			Fraction	-0.02	7.14E-01		129
		Tacrolimus level in blood	Absolute UCr adj.	0.25	4.77E-02	*	32
			Fraction	0.04	7.70E-01		32
**LT**	Plasma	ALAT	Absolute	0.64	2.17E-21	*	103
			Fraction	0.49	3.52E-13	*	103
		ASAT	Absolute	0.59	3.15E-18	*	102
			Fraction	0.45	2.39E-11	*	102
		CRP	Absolute	0.26	4.24E-04	*	88
			Fraction	0.22	3.25E-03	*	88
		Leucocytes in blood	Absolute	0.19	3.46E-03	*	104
			Fraction	0.23	7.13E-04	*	104
		Tacrolimus level in blood	Absolute	-0.24	2.53E-03	*	72
			Fraction	-0.25	1.89E-03	*	72

*All samples from the early, stable, and nonstable groups are combined. The correlations were performed using Kendall's method. *indicates a p-value<0.05.*

Figure S10: Plasma ddcfDNA in KT correlations to other biomarkers. Kendall's tau correlation was done for each patient category.

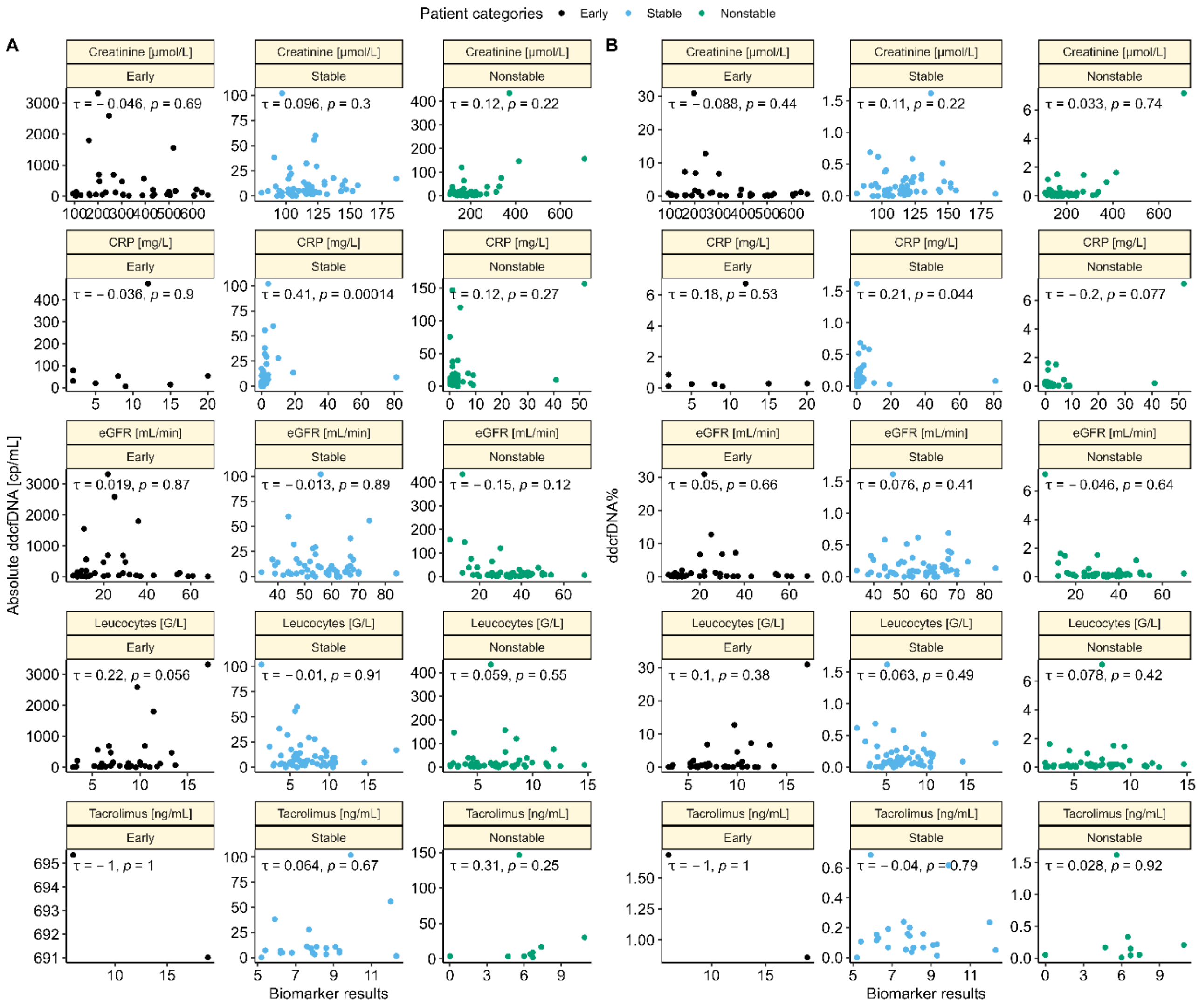

Figure S11: Urine ddcfDNA in KT correlations to other biomarkers. The correlations were performed for each subgroup separately with Kendall's tau.

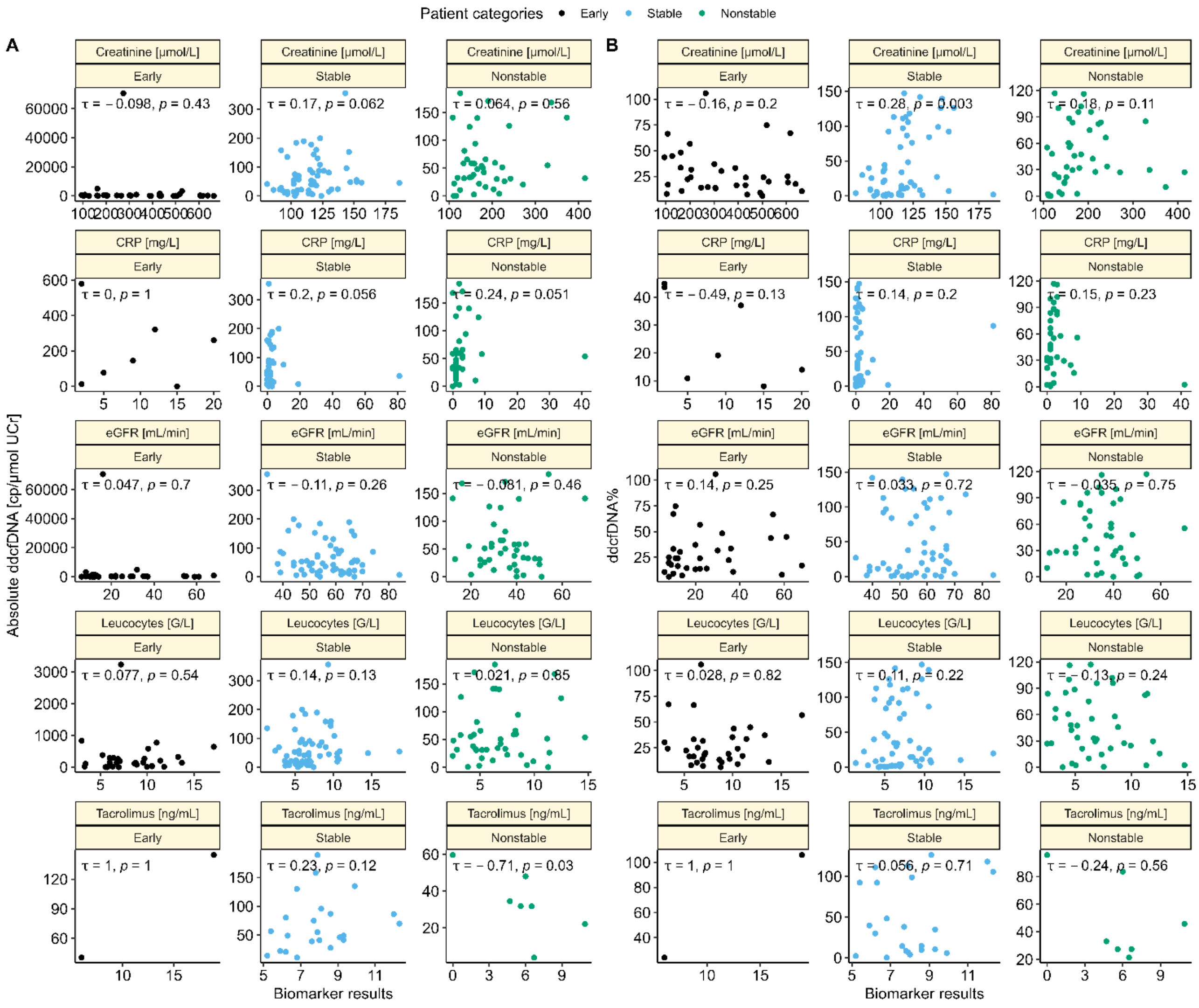

Supplement: Supplementary file 1 [file Supplementaryfile1.pdf]
